# Developing and evaluating an instrument to assess perceptions of an entry-level physician associate doctoral degree

**DOI:** 10.1186/s12909-022-03668-1

**Published:** 2022-08-13

**Authors:** Hyun-Jin Jun, Karen L. Gordes, Shani Fleming, Violet Kulo, James F. Cawley, Gerald Kayingo

**Affiliations:** 1 Health Professions Education, Physician Assistant Leadership and Learning Academy, Graduate School University of Maryland Baltimore, Baltimore, MD USA; 2 UMB Physician Assistant Program, Physician Assistant Leadership and Learning Academy, Graduate School University of Maryland Baltimore, Baltimore, MD USA; 3 Physician Assistant Leadership and Learning Academy, Graduate School University of Maryland Baltimore, Baltimore, MD USA

**Keywords:** Health professions education, Measures, Instrument, Perceptions, Entry-level doctoral degree, Physician assistants/associates

## Abstract

**Background:**

Most health professions in the United States have adopted clinical or practice doctorates, sparking an ongoing debate on whether physician assistants/associates (PAs) should transition from a master’s to a doctorate as the terminal degree for the profession. Although more studies are anticipated, there is no validated instrument assessing perceptions of various stakeholders regarding an entry-level PA doctoral degree. The objective of this study was to develop and evaluate a novel self-report measure to assess perceptions of an entry-level PA doctoral degree.

**Methods:**

A multifaceted, mixed-methods approach was adopted. Based on a comprehensive literature review of the doctoral transition experiences in other health professions, an initial version of *perceptions of an entry-level terminal PA doctoral degree scale* (PEDDS) was generated. This scale was pilot tested with a group of PA faculty, students, and clinicians. Then, a cross-sectional survey consisting of 67 items was conducted with a national random sample of practicing PAs and PA students. Additionally, semi-structured interviews were conducted to ensure the validity of PEDDS. A principal component analysis (PCA) was conducted to reduce the number of items and reveal the underlying structure of PEDDS.

**Results:**

The PCA confirmed 10 factors of PEDDS consisting of 53 items as the best-fit factor structure with adequate internal consistency of subscales. Those factors include a) expected positive impact on the PA profession, b) expected impact on prerequisites, (c) expected impact on the student preparedness as PA faculty and educators, (d) expected impact on the student preparedness as clinicians, (e) expected impact on accreditation and certification, (f) expected impact on curriculum, (g) expected impact on PA educators, (h) expected positive impact on diversity, (i) expected negative impact on the PA profession, and (j) expected impact on the student competency.

**Conclusions:**

The present study highlights the need to develop valid and reliable measurements to assess perceptions regarding the transition to the entry-level doctorate across health professions. This study could be used to guide further discussion of the entry-level doctorates for PAs and other health professions by bridging the gap of existing literature related to valid, reliable, and standardized measures on this topic.

**Supplementary Information:**

The online version contains supplementary material available at 10.1186/s12909-022-03668-1.

## Background

In the last two decades, there has been a growing trend toward entry-level doctoral degrees in various health professions in the United States (U.S.) [1, 2]. Entry-level clinical doctorates have been adopted in the fields of pharmacy, physical therapy, and advanced practice nursing [3–5]. To date, a master’s degree is considered the terminal degree and the minimum entry-level qualification in the physician assistant/associate (PA) profession, but there has been ongoing debate regarding an entry-level doctoral degree for PAs since mid-90’s [6, 7]. In 2009, a formal PA Doctoral Summit was convened by national PA organizations (i.e., American Academy of PAs [AAPA], Physician Assistant Education Association [PAEA]) to discuss the future of doctoral education for PAs. After obtaining input from a wide range of stakeholders, summit participants recommended against an entry-level doctoral degree for PAs but encouraged PAs to explore various options for post-professional training [8]. Over time, there has been growing interest and research exploring perceptions of various stakeholders regarding doctoral education for PAs. Using a randomized sample of 1500 U.S. PAs, a cross-sectional study investigating perceptions of the entry-level doctoral degree of practicing PAs revealed that the majority of respondents did not support moving toward an entry-level doctorate for the PA profession [9]. Similar findings were also reported in subsequent studies [10–14]. Consequently, the national PA organizations such as AAPA and PAEA have opposed several motions to adopt the entry-level terminal doctoral degree for PAs in part due to increased student debt burden and the potential adverse impact on diversity of the workforce [15].

As the health care milieu continues to change, the quest for doctoral education for PAs has continued at various levels. As of 2022, about 11 institutions have established post-professional programs such as the Doctor of Medical Science [6, 7, 12, 16, 17]. However, the value, risks, and benefits are still subject to discussion, and the PA entry-level doctoral credential debate continues. Recently investigated the potential risks and benefits of the entry-level doctoral degree for PAs. The results from this study were mixed, with some people advocating for the entry-level doctorate, whereas others against it [12, 16]. Similarly, the AAPA has undertaken a study assessing current perceptions and given guidance to the professions [18]. In May 2021, the subject of the entry-level doctorate for PAs was amongst the hotly discussed topics in the House of Delegates. Taken together, the research and various discussions call for further investigation to guide future discussion of the entry-level doctorates for PAs. Over a dozen scholarly documents have been produced in the past year alone on this topic. As we expect that more studies will be conducted in this field, it is essential for researchers to have a reliable, valid, and replicable/reproducible instrument to conduct research on terminal doctoral degrees in health professions. To our knowledge, there is no valid and reliable instrument assessing perceptions of various stakeholders regarding an entry-level PA doctoral degree. This study aimed to develop and evaluate a novel self-report measure to assess perceptions of an entry-level PA doctoral degree.

## Methods

### Study procedures and participants

**Fig. 1:**
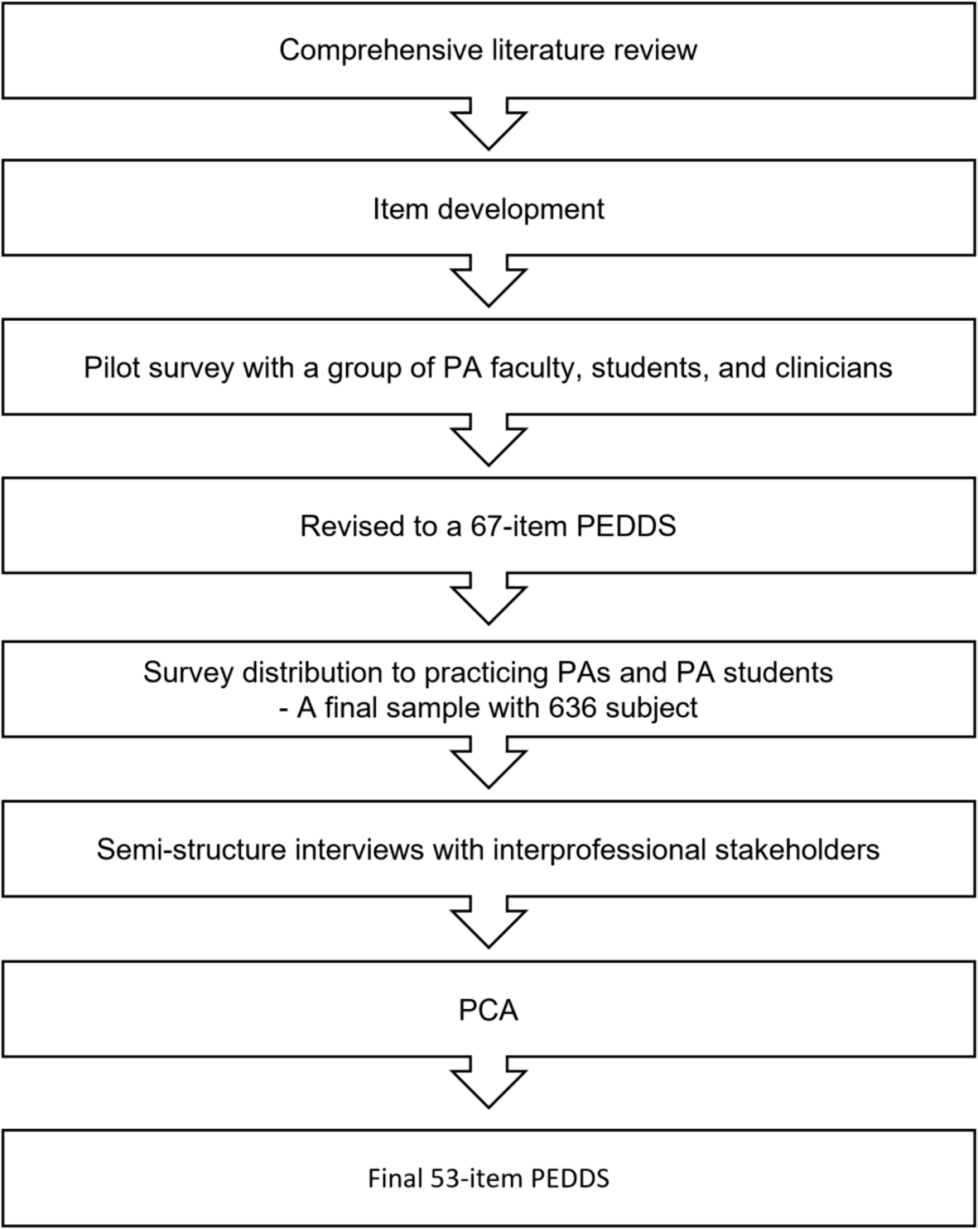
Scale development methodology

A multifaceted, mixed-methods approach was used to develop and test an instrument to measure perceptions of an entry-level PA doctoral degree (Fig. 1). The data used in this study is part of large study exploring perceived benefits and impacts of an entry-level PA doctoral degree. Detailed study and data collection procedures have been previously described [12]. This study was approved by the Institutional Review Board (IRB) of the University of Maryland Baltimore. Informed consent was obtained from all participants.

### Step 1: literature review

A comprehensive review of the existing literature as the ground of the instrument development was completed to investigate the impact of the transitions of health professions doctoral programs including nursing, pharmacy, and physical therapy. Articles were searched in June 2020 through electronic databases including CINAHL (Cumulative Index to Nursing and Allied Health Literature), Cochrane Database of Systematic Reviews, Medline, and PubMed based on inclusion and exclusion criteria. We focused on exploring risk, benefits, outcomes for the doctoral transition, and related political and regulatory factors. Based on the literature review, research team members who have extensive experience in PA education identified overarching themes and key concepts to capture perceived benefits and risks of the doctoral transition. Those themes and concepts were evaluated for content validity and consistency by research team members, and any discrepancy was discussed until consensus was reached. Overarching themes included degree evolution, political and regulatory factors that promoted transition, and the impact of the transition on faculty, students, profession, and education curricula changes [6]. Key concepts within the overarching themes of perceived benefits and risks prior to their transition as well as the impact after their transition identified from literature review were used to generate the questionnaires for both the quantitative survey and qualitative interviews.

### Step 2: item development and online cross-sectional survey

The *perceptions of an entry-level PA doctoral degree scale* (PEDDS) were developed for the use of a cross-sectional survey. It was based primarily on the empirically derived set of constructs identified in prior research [2, 6], which would capture the breadth of the potential impact of the doctoral transitions in health professions. The themes for PEDDS include the benefits and risk, the impact on the PA profession/PA educator/clinical training sites/scope of practice, outcomes, and expected impact to students’ preparedness/competency/curriculum/prerequisites. As a preliminary step, PEDDS was pilot tested with a diverse group of PA faculty, students, and practicing clinicians to achieve face validity and reliability of the instrument. A convenience sample of participants across the country was recruited to minimize potential bias. Based on their recommendations, PEDDS was revised into 67 items using a five-point Likert scale (1 = *strongly disagree* to 5 = *strongly agree* or 1 = *extremely unlikely to* 5 = *extremely likely*) to assess participant’s self-reported perspectives on the risks, benefits, and impact of transitioning to an entry-level PA doctoral degree.

A revised survey was distributed to a random sample of practicing PAs and PA students across the U.S. in 2020 through Qualtrics, a web-based software/platform for creating and distributing online surveys. The online survey was distributed to 1368 participants (926 practicing PAs and 442 PA students) through AAPA’s PA Observations Service (a service that connects students, researchers, and organizations to PAs who wish to share their experiences/perspectives) and the Maryland Academy of Physician Assistants listserv (MdAPA) that has about 5500 members. A total of 476 responses (35% response rate) and an additional 160 responses were received from AAPA’s PA Observations Service and MdAPA, respectively. A final sample included 636 subjects who consented and completed the survey. It is recommended to have at least 150 cases or 5 to 10 cases per variable for PCA, which yields to have between 335 to 670 samples for the current study [19].

### Step 3: semi-structured interviews

Using a purposive sampling, we conducted semi-structured interviews with various interprofessional stakeholders to collect information on attitudes, opinions, and experiences related to entry-level health profession doctoral degrees. 38 participants who participated in the semi-structured interviews included 19 PA association leaders and members (50%), nine PA program directors and faulty (24%), six non-PA academic leaders (16%), two physicians (5%), and two employers (5%). Sample characteristics were previously described in detail [12]. Based on the findings from the interprofessional literature review, a semi-structured interview guide consisting of 11 items was developed to assess feasibility, benefits, and risks as well as the impact of an entry-level PA doctorate degree. First, the same eight open-ended questions were asked to all stakeholders (e.g., “what do you see are the benefits and risks of conferral of a doctorate degree for entry-level PAs?”, “what do you foresee the impact of doctorate PA programs to be on the PA profession? Do you anticipate changes in scope of practice? Impact on diversity of PA workforce? PA educators? Any new roles for PA with an advanced degree?”). Then, three open-ended questions specific to each stakeholder group were asked (e.g., “how do you think your program would be affected should this transition occur?”, “What institutional and faculty qualifications are needed to meet the demands of a doctorate program?”). The results of the semi-structured interviews were triangulated with the cross-sectional survey data to ensure content validity of PEDDS.

#### Statistical analysis

For the analytical purpose, a five-point Likert scale was coded into three categories as follows: 0 = disagree (combining strongly and somewhat disagree), 1 = neutral, and 2 = agree (combining strongly and somewhat agree) for questions regarding the level of agreement; 0 = unlikely (combining extremely and somewhat unlikely), 1 = neutral, and 2 = likely (combining extremely and somewhat likely) for those regarding the level of likelihood. All analyses were conducted using SPSS version 28 for Windows [20]. Descriptive analyses were performed to understand the sample characteristics and the main study variables. A principal component analysis (PCA) was conducted to reduce the number of items and to reveal the underlying structure of PEDDS. Factorability of the 67 items were assessed through several preliminary tests related to the assumption checking. None of the assumptions for the PCA was violated. First, data were initially screened for missing data and univariate outliers across each item. There were no significant outliers and a few missing data across items. Missing data were handled using listwise deletion due to the low frequency of missing values (ranging from 0.2 to 0.9%). Second, the normality assumption was confirmed by assessing the skewness. Third, Pearson correlation coefficients indicated that each item was correlated at least to one other item with a linearity of variables, ranging from .08 to .79. Fourth, the Kaiser-Meyer-Olkin (KMO) measure for sampling adequacy was .97, indicating the sampling adequacy was excellent to run PCA (> .60) [21, 22]. Lastly, Bartlett’s test of sphericity was significant (*χ*^*2*^ (2211) = 29,815.73, *p* < .001), indicating adequate correlations between variables to compress the data into a smaller number of components in a meaningful way [23]. For the main analysis, PCA was conducted with oblique (a direct oblimin [nonorthogonal]) rotation techniques of the factor loading (the correlation coefficient between the variable and the factor) matrix, assuming factor to be correlated based on the literature [23]. Communalities (the proportion of each variable’s variance that can be explained by the factors) ranged from .39 to .86, suggesting that there is common variance across all items. Factors with eigenvalues (values that represent the amount of the common variance explained by a factor) greater than 1 [24], and factor loadings with a cut-off point of .40 or above [25, 26] were retained. Along with the PCA, exploratory factor analysis (EFA) was conducted. The results between PCA and EFA were similar, but the PCA better presented components without cross-loading items. Therefore, we adopted the PCA for a data reduction method to figure out the optimal number of components and items for each component.

## Results

**Fig. 2:**
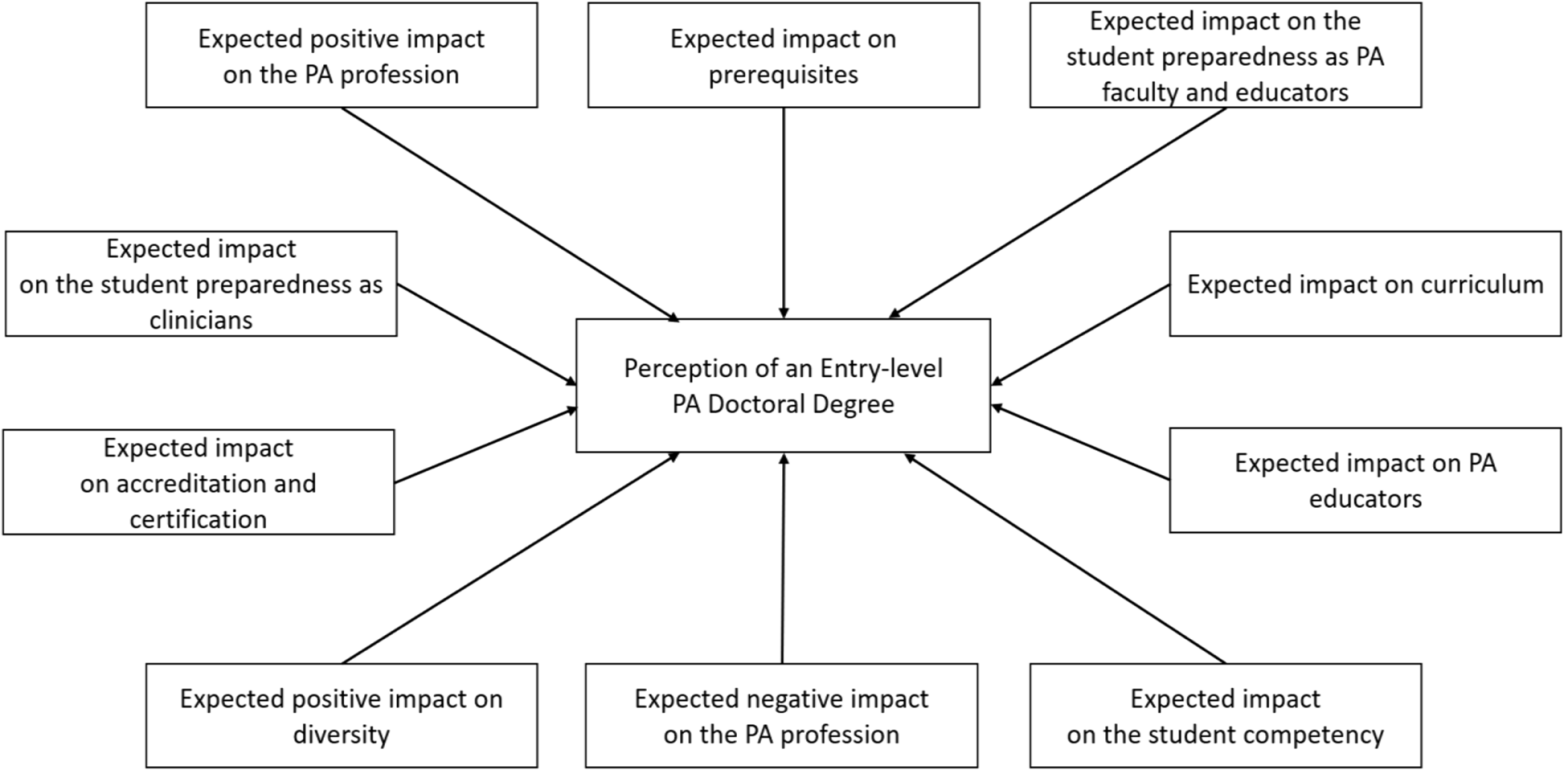
Proposed model for perceptions of an entry-level PA doctoral degree

The sample characteristics of this study are presented in Table 1. The results of PCA are presented in Table 2. For item deduction, 14 items with a factor loading of less than .4 or irrelevant items from the theoretically planned factor were excluded. The 53 items with a primary factor loading of at least .4 or above were retained for the final version of PEDDS. Factorial validity assessed by the eigenvalues and scree plots yielded 10 factors of PEDDS as the best-fit factor structure, accounting for 66.2% of variance (Fig. 2).

**Table 1:** Description of sample characteristics (*N* = 636)

	*n*^a^	*%*
Sex		
Female	433	68.5
Male	199	31.5
Hispanic/Latinx		
Yes	47	7.4
No	584	92.6
Race		
White	523	82.2
Black/African American	39	6.1
American Indian/Alaska Native	6	0.9
Asian	41	6.4
Native Hawaiian/Pacific Islander	2	0.3
Multiracial	18	2.8
Others	19	3.0
Educational attainment		
Associate degree	2	.3
Bachelor’s degree	97	15.3
Master’s degree	475	74.9
Doctorate degree	60	9.5
Occupation^b^		
Student	109	17.2
PA clinician	524	82.8
Length of practice as PA		
Current student	107	16.9
< 5 years	172	27.1
5–10 years	148	23.3
11–25 years	169	26.7
> 25 years	37	5.8
Non-PA	1	.2

^a^Variations existed in the number of respondents by question

^b^Some of the PA clinicians reported additional professional roles such as faculty and administrators but they were all counted as PA clinicians

**Table 2:** Factor loadings and communalities from the principal components analysis of the PEDDS

Components	Item	Factor										Communality
		1	2	3	4	5	6	7	8	9	10	
Expected positive impact on the PA profession	An entry-level PA doctoral degree will have the following impact on the PA Profession - Enhance billing and reimbursement opportunities.	.87										.64
	An entry-level PA doctoral degree will have the following impact on PA scope of practice and outcomes - Promote PA practice Autonomy	.82										.71
	An entry-level PA doctoral degree will have the following impact on the PA Profession - Enable parity with other professions.	.80										.71
	An entry-level PA doctoral degree will have the following impact on the PA Profession - Enhance the competitive advantage (edge).	.80										.78
	An entry-level PA doctoral degree will have the following impact on PA scope of practice and outcomes - Enhance billing and reimbursement opportunities.	.80										.79
	An entry-level PA doctoral degree will have the following impact on PA scope of practice and outcomes - Enable PAs to practice at the top of their license.	.79										.78
	An entry-level PA doctoral degree will have the following impact on the PA Profession - Advance public recognition.	.75										.73
	An entry-level PA doctoral degree will have the following impact on PA scope of practice and outcomes - Enhance Optimal Team Practice (OTP).	.65										.69
	How likely an entry-level PA doctoral degree will enhance scope of practice.	.64										.62
	An entry-level PA doctoral degree will have the following impact on PA scope of practice and outcomes - Increase access, quality, cost effective care.	.55										.70
	An entry-level PA doctoral degree will have the following impact on PA scope of practice and outcomes - Increase patient satisfaction.	.54										.67
Expected impact on prerequisites	How likely will the following prerequisites be impacted if the PA profession adopts an entry-level PA doctoral degree? – GPA.		.87									.76
	How likely will the following prerequisites be impacted if the PA profession adopts an entry-level PA doctoral degree? – GRE.		.77									.61
	How likely will the following prerequisites be impacted if the PA profession adopts an entry-level PA doctoral degree? - Pre-requisite courses (statistics, basic sciences).		.76									.60
	How likely will the following prerequisites be impacted if the PA profession adopts an entry-level PA doctoral degree? - Prior clinical experience.		.66									.47
	How likely will the following prerequisites be impacted if the PA profession adopts an entry-level PA doctoral degree? - Entrance examination (ie PCAT).		.65									.49
Expected impact on the student preparedness as PA faculty and educators	As compared to a master’s degree, I believe an entry-level PA doctoral degree education will better prepare students in the following areas - Academia/Teaching skills.			−.85								.69
	As compared to a master’s degree, I believe an entry-level PA doctoral degree education will better prepare students in the following areas – Administration.			−.80								.70
	As compared to a master’s degree, I believe an entry-level PA doctoral degree education will better prepare students in the following areas - Program & Policy development.			−.60								.66
	As compared to a master’s degree, I believe an entry-level PA doctoral degree education will better prepare students in the following areas - Research skills.			−.58								.53
	As compared to a master’s degree, I believe an entry-level PA doctoral degree education will better prepare students in the following areas – Leadership.			−.40								.60
Expected impact on the student preparedness as clinicians	As compared to a master’s degree, I believe an entry-level PA doctoral degree education will better prepare students in the following areas - Clinical practice skills.				−.82							.77
	As compared to a master’s degree, I believe an entry-level PA doctoral degree education will better prepare students in the following areas - The demands of working as a clinician.				−.78							.79
	As compared to a master’s degree, I believe an entry-level PA doctoral degree education will better prepare. Students in the following PA competency areas - Patient-centered practice knowledge (Medical Knowledge).				−.75							.75
	As compared to a master’s degree, I believe an entry-level PA doctoral degree education will better prepare students in the following areas - The readiness for team based and collaborative patient care.				−.70							.79
	As compared to a master’s degree, I believe an entry-level PA doctoral degree education will better prepare students in the following areas - Being up-to-date in new or innovative evidence-based practice.				−.68							.70
	As compared to a master’s degree, I believe an entry-level PA doctoral degree education will better prepare students in the following areas - Use of theory in practice.				−.56							.61
	As compared to a master’s degree, I believe an entry-level PA doctoral degree education will better prepare students in the following PA competency areas - Cultural humility.				−.44							.68
Expected impact on accreditation and certification	How likely an entry-level PA doctoral degree will - Change the certification process.					.87						.84
	How likely an entry-level PA doctoral degree will - Change the recertification process.					.84						.78
	How likely an entry-level PA doctoral degree will - Change accreditation standards.					.74						.61
Expected impact on curriculum	An entry-level PA doctoral degree will have the following impact on curriculum - Require no additional content.						.76					.63
	An entry-level PA doctoral degree will have the following impact on curriculum - Require new content.						−.73					.67
	An entry-level PA doctoral degree will have the following impact on curriculum - Require significant change.						−.60					.52
	Some people believe that the current number of credits, depth, and breadth of PA training offered in most PA programs to date are sufficient for a doctoral degree. How strongly do you agree or disagree with this statement?						.53					.39
Expected impact on PA educators	An entry-level PA doctoral degree will have the following impact on PA educators: - PA educator competencies will change.							.91				.76
	An entry-level PA doctoral degree will have the following impact on PA educators: - PA educator credentials will change.							.84				.81
	An entry-level PA doctoral degree will have the following impact on PA educators - PA educator shortage will worsen.							.43				.62
Expected positive impact on diversity	How likely an entry-level PA doctoral degree will - Shift practice setting to urban, rural or underserved locations.								.81			.69
	How likely an entry-level PA doctoral degree will - Shift practice setting to primary care.								.79			.62
	How likely an entry-level PA doctoral degree will - Increase diversity.								.66			.70
	An entry-level PA doctoral degree will have the following impact on the PA Profession - Increase diversity.								.54			.71
	An entry-level PA doctoral degree will have the following impact on the PA Profession - Increase enrollment and demand.								.42			.63
Expected negative impact on the PA profession	An entry-level PA doctoral degree will have the following impact on the PA Profession - Increase the cost of PA education.									.61		.42
	An entry-level PA doctoral degree will have the following impact on PA scope of practice and outcomes - Limit PA flexibility working across various specialties.									.58		.42
	How likely an entry-level PA doctoral degree will - Confuse the patient.									.53		.52
	An entry-level PA doctoral degree will negatively impact the PA-Physician relationship.									.44		.53
Expected impact on the student competency	As compared to a master’s degree, I believe an entry-level PA doctoral degree education will better prepare students in the following PA competency areas - Professional and legal aspects of health care.										.72	.79
	As compared to a master’s degree, I believe an entry-level PA doctoral degree education will better prepare students in the following PA competency areas - Health care finance and systems.										.69	.72
	As compared to a master’s degree, I believe an entry-level PA doctoral degree education will better prepare students in the following PA competency areas - Society and population health.										.64	.73
	As compared to a master’s degree, I believe an entry-level PA doctoral degree education will better prepare students in the following PA competency areas - Health literacy and communication.										.61	.72
	As compared to a master’s degree, I believe an entry-level PA doctoral degree education will better prepare students in the following PA competency areas - Interprofessional collaborative practice and leadership.										.58	.69
	As compared to a master’s degree, I believe an entry-level PA doctoral degree education will better prepare students in the following PA competency areas - Ongoing Professional development.										.42	.60

Initial eigenvalues indicated that the first factor (11 items), *expected positive impact on the PA profession*, explained 32.4% of variance with an eigenvalue of 17.15. Example items loaded onto the first factor include: a) An entry-level PA doctoral degree will have the impact on the PA Profession in enhancing billing and reimbursement opportunities, b) An entry-level PA doctoral degree will have the impact on PA scope of practice and outcomes - in promoting PA practice autonomy, c) An entry-level PA doctoral degree will have the impact on PA scope of practice and outcomes in- enabling PAs to practice at the top of their license, d) An entry-level PA doctoral degree will have the impact on the PA Profession in advancing public recognition, and e) An entry-level PA doctoral degree will have the impact on PA scope of practice and outcomes in increasing access, quality, and cost-effective care. Excellent internal consistency for first factor items was found with this sample (*α* = .96). Composite scores were created to explore the descriptive statistics (Table 3), indicating that higher scores for items in the first factor suggest more positive perceptions for the entry-level doctoral degree on the PA profession (*M* = 22.08, *SD* = 12.20, range = 0–42).

**Table 3:** Descriptive statistics for PEDDS

Subscale	Number of items	M (SD)	Skewness	Kurtosis	Cronbach’s alpha
Expected positive impact on the PA profession	11	22.08 (12.20)	−.20	−.90	.96
Expected impact on prerequisites	5	6.53 (3.01)	−.73	−.41	.81
Expected impact on the student preparedness as PA faculty and educators	5	6.98 (3.12)	−.94	−.14	.84
Expected impact on the student preparedness as clinicians	7	5.64 (4.95)	4.11	−1.23	.93
Expected impact on accreditation and certification	3	4.59 (1.87)	−1.17	.26	.82
Expected impact on curriculum	4	24.16 (3.01)	−.69	.59	.63
Expected impact on PA educators	3	9.00 (2.40)	−.72	.34	.67
Expected positive impact on diversity	5	4.05 (3.43)	.82	.02	.82
Expected negative impact on the PA profession	4	8.29 (2.45)	−.61	−.13	.51
Expected impact on the student competency	6	3.53 (2.28)	−.41	−1.31	.86

The second factor consisting of 5 items referred to *expected impact on prerequisites*, including the likelihood of the impact on prerequisites (i.e., GPA, GRE, prerequisites courses, prior clinical experience, entrance examination). The second factor accounted for 8.4% of variance with an eigenvalue of 4.4. Higher sum scores of items in this scale indicate greater likelihood of being impacted on prerequisites with a transition to the doctoral degree (*M* = 6.53, *SD* = 3.01, range = 0–10). Good internal consistency for second factor items was found with this sample (*α* = .81).

The remaining eight factors with eigenvalues over 1 collectively accounted for 25.4% of the variance. The third (5 items, 5.0% of variance) and the fourth factors (7 items, 4.3% of variance) assessed *expected impact on the student preparedness as PA faculty and educators* (academia/teaching skills, administration, program and policy development, research skills, leadership)*,* and *expected impact on the student preparedness as clinicians* (e.g.*,* clinical practice skills, patent-centered practice knowledge, the readiness for team-based and collaborative patient care), respectively. Both of these factors presented good internal consistency with this sample (*α* = .84 for third and .93 for fourth factors). *Expected impact on accreditation and certification* (*M* = 4.59, *SD* = 1.87, range = 0–6) and *curriculum* (*M* = 24.16, *SD* = 3.01, range = 12–29) were presented in the fifth (3 items, 3.4% of variance) and sixth (4 items, 3.2% of variance) factors, respectively. The seventh (3 items, 2.7% of variance) and ninth (4 items, 2.3% of variance) factors assessed *expected impact on PA educator* (*M* = 9.00, *SD* = 2.40, range = 0–6) and *expected negative impact on the PA profession (M* = 8.29, *SD* = 2.45, range = 1–12). *Expected positive impact on diversity* (5 items, 2.6% of variance) and *student competency* (6 items, 2.1% of variance) were assessed in the eighth (*M* = 4.05, *SD* = 3.43, range = 0–14) and 10th (*M* = 3.53, *SD* = 2.28, range = 0–6) factors. The overall internal consistency ranged from .51 to .96. Except for the subscale of *expected negative impact on the PA profession* (*α* = .51), the internal consistencies for all subscales with this sample were moderate or higher, ranging from .63 to .96 [27].

## Discussion

The present study sought to examine the underlying structure of a novel self-report measure regarding perceptions of an entry-level PA doctoral degree using PCA. We found that a 10-factor solution accounted for over three-fifth of the variance, and all those factors presented adequate internal consistency as separate subscales. The first factor composed of 11 items assessed expected positive impact on the PA profession, particularly in terms of amplifying billing and reimbursement opportunities, PA practice autonomy, optimal team practice [13], the competitive advantage, public recognition, scope of practice, access/quality/cost effective care, and patient satisfaction. Higher scores indicate greater perceptions regarding the positive impact of the entry-level doctorate on the PA profession. For the ninth factor, expected negative impact on the PA profession (4 items) has been identified, which addresses possibilities for increasing the cost of education, limiting flexibility working across various specialties, confusing the patients, and causing dysfunctional relationships between PAs and physicians. Four subscales assess educational aspects regarding the transition to the doctoral degree, such as expected impact on prerequisites (5 items), curriculum (4 items), accreditation and certification (3 items), and PA educators (3 items). Other factors focus on the student preparedness as PA faculty and educators (5 items), the student preparedness as clinicians (7 items), and the student competency (6 items). It is noteworthy that 5-item expected positive impact on diversity subscale has been confirmed, which includes shifting practice settings to urban, rural or underserved locations and to primary care and increasing enrollment and demand. The internal consistencies for the subscales of PEDDS were moderate or higher, ranging from .63 to .96, notwithstanding the low Cronbach’s alpha for the subscale of expected negative impact on the PA profession (*α* = .51). The findings of the current study indicate that PEDDS would be a useful instrument to assess a wide range of perspectives or perceptions regarding the transition to the entry-level doctorate.

A body of research has been conducted to assess perceptions regarding an entry-level doctorate in various health professions in past decades. However, we are unaware of any valid and reliable measurement tool utilized in the field of health professions. Additionally, most of the studies have focused on a certain sample or group (e.g., students, program directors) in this area although a transition to the entry-level doctoral degree would have a wide range of impact to various stakeholders and aspects of the profession. For instance, Swanchak and colleagues (2011) conducted research to explore perceptions of transitioning the entry-level degree for PAs to a clinical doctorate with a sample of 1996 PA students from 30 PA programs with 15 items using a 5-point Likert scale. Despite high internal reliability of the items, no standardized or valid instrument was used in the study, and the study only focused on the students’ perspectives [28]. Similarly, Menezes et al. (2015) investigated the attitudes of PA students toward a clinical doctorate and related impacts with 1658 PA students from 53 PA programs [29]. The survey instrument was pilot tested but not validated. Coplan et al. (2009) conducted mixed-methods research to explore the opinions regarding an entry-level clinical doctorate among 152 PA program medical directors using a 16-item, non-validated survey [11]. Recently, a study conducted by Brown and colleagues (2020) addressed the potential impact of an entry-level doctorate on PAs and PA faculty and programs among 712 PA educators using a 32-item survey that has not been validated [16]. Although Muma et al. (2011) included representative samples of physicians, PAs, and PA faculty to compare perceptions regarding the entry-level doctoral education, a non-validated instrument seemed to be used [14]. Similar issues with using non-validated instruments in this topic have been found in other health professions research such as nursing [30], pharmacy [31, 32], physical therapy [33], and occupational therapy [34]. Given the importance of this topic, this study highlights the need to develop valid and reliable measurements to assess the various perceptions regarding the transition to the entry-level doctorate across health professions.

To the best of our knowledge, PEDDS is the first self-report instrument to assess perceptions regarding the entry-level PA doctoral degree. The PEDDS was a key in this investigation which used a multi-prong, mixed-methods approach, involving interprofessional literature review, cross-sectional survey, and semi-structured interviews to capture stakeholders’ views on and impact of transitioning to an entry-level PA doctorate. The survey instrument was beta tested, and recommendations were used to refine the survey prior to distribution to study participants. Using systematic approaches for scale development, this study includes perspectives and insights of various stakeholders, indicating potential multifaceted impact pertaining to the transition to an entry-level doctorate. The strength of this study includes the large sample size using a probability sampling, which contributes to validating the results. This study could be used to guide further discussion of the entry-level doctorates for PAs and other health professions by bridging the gap of existing literature related to valid, reliable, and standardized measures in this topic.

Despite the significance and strengths of the present study, there are some limitations. In terms of sampling, we were not able to track how many PA students and clinicians were included in the overall sampling list. This made it difficult to provide detailed response rates for a certain subgroup, factor, or characteristics. Due to small cases in a certain subgroup (e.g., faculty, administrator, those with doctoral degree), they were not broken down for the main analyses to reduce potential response bias. However, no significant differences were found with and without those subgroups. A low response rate from AAPA’s PA observations Service could negatively affect the reliability and validity of results in this study. This cross-sectional study was conducted by distributing the survey one time; hence, test-retest reliability has not been established. Future research should redistribute the survey to ensure the reliability of measures on repeated administration. The internal consistency of the subscale of expected negative impact on the PA profession was low, and this may be partly due to the small number of items in this subscale [35]. Based on a preliminary study of a novel measure developed in the present study, future research should conduct a confirmatory factor analysis (CFA) to validate factor structure of the constructs identified in this study. Although generalization to other countries is limited because the present study was conducted in the U.S. context, this study could be replicated by diversifying the professions and countries to produce an instrument that could be used in other contexts and with other professions.

## Conclusions

A novel self-report measurement instrument, PEDDS, was developed using a multi-prong, mixed-methods approach, involving an interprofessional literature review, cross-sectional surveys, and semi-structured interviews to capture stakeholders’ views on the impact of transitioning to an entry-level PA doctorate. This study will be useful in guiding further discussion of the entry-level doctorates for PAs and other health professions by bridging the gap of existing literature related to valid, reliable, and standardized measures in this topic. This instrument has potential to be adopted by other health professions considering a shift to entry-level doctoral education.

## Supplementary Information

**Additional file 1: Supplementary material**. Survey items by response options.

## Data Availability

The datasets used and/or analyzed during the current study are available from the corresponding author on reasonable request.

## Abbreviations

AAPA: American Academy of Physician Assistants
MdAPA: Maryland Academy of Physician Assistants listserv
PA(s): Physician Assistant/Associate
PAEA: Physician Assistant Education Association
PEDDS: Perceptions of an Entry-level PA Doctoral Degree Scale
U.S.: United States
PCA: Principal component analysis
