# Supplementary material for: Developing and evaluating an instrument to assess perceptions of an entry-level physician associate doctoral degree
Source: BMC Med Educ. 2022 Aug 13;22:617. doi: 10.1186/s12909-022-03668-1 (PMC9375372; doi:10.1186/s12909-022-03668-1)
Supplement: Supplementary file 1 — Additional file 1: Supplementary material. Survey items by response options. [file 12909_2022_3668_MOESM1_ESM.docx]

**Supplementary material** Survey items by response options

| Item |
| --- |
| Original response options: *from strongly Disagree to strongly agree* |
| In your opinion, an entry-level PA doctoral degree will have the following impact on the PA Profession - Enhance billing and reimbursement opportunities. |
| In your opinion, an entry-level PA doctoral degree will have the following impact on PA scope of practice and outcomes - Promote PA practice Autonomy |
| In your opinion, an entry-level PA doctoral degree will have the following impact on the PA Profession - Enable parity with other professions. |
| In your opinion, an entry-level PA doctoral degree will have the following impact on the PA Profession - Enhance the competitive advantage (edge). |
| In your opinion, an entry-level PA doctoral degree will have the following impact on PA scope of practice and outcomes - Enhance billing and reimbursement opportunities. |
| In your opinion, an entry-level PA doctoral degree will have the following impact on PA scope of practice and outcomes - Enable PAs to practice at the top of their license. |
| In your opinion, an entry-level PA doctoral degree will have the following impact on the PA Profession - Advance public recognition. |
| In your opinion, an entry-level PA doctoral degree will have the following impact on PA scope of practice and outcomes - Enhance Optimal Team Practice (OTP). |
| In your opinion, an entry-level PA doctoral degree will have the following impact on PA scope of practice and outcomes - Increase access, quality, cost effective care. |
| In your opinion, an entry-level PA doctoral degree will have the following impact on PA scope of practice and outcomes - Increase patient satisfaction. |
| In your opinion, an entry-level PA doctoral degree will have the following impact on curriculum - Require no additional content. |
| In your opinion, an entry-level PA doctoral degree will have the following impact on curriculum - Require new content. |
| In your opinion, an entry-level PA doctoral degree will have the following impact on curriculum - Require significant change. |
| In your opinion, an entry-level PA doctoral degree will have the following impact on PA educators: - PA educator competencies will change. |
| In your opinion, an entry-level PA doctoral degree will have the following impact on PA educators: - PA educator credentials will change. |
| In your opinion, an entry-level PA doctoral degree will have the following impact on PA educators - PA educator shortage will worsen. |
| In your opinion, an entry-level PA doctoral degree will have the following impact on the PA Profession - Increase diversity. |
| In your opinion, an entry-level PA doctoral degree will have the following impact on the PA Profession - Increase enrollment and demand. |
| In your opinion, an entry-level PA doctoral degree will have the following impact on the PA Profession - Increase the cost of PA education. |
| In your opinion, an entry-level PA doctoral degree will have the following impact on PA scope of practice and outcomes - Limit PA flexibility working across various specialties. |
| In your opinion, an entry-level PA doctoral degree will negatively impact the PA-Physician relationship. |
| As compared to a master’s degree, I believe an entry-level PA doctoral degree education will better prepare students in the following areas - Academia/Teaching skills. |
| As compared to a master’s degree, I believe an entry-level PA doctoral degree education will better prepare students in the following areas – Administration. |
| As compared to a master’s degree, I believe an entry-level PA doctoral degree education will better prepare students in the following areas - Program & Policy development. |
| As compared to a master’s degree, I believe an entry-level PA doctoral degree education will better prepare students in the following areas - Research skills. |
| As compared to a master’s degree, I believe an entry-level PA doctoral degree education will better prepare students in the following areas – Leadership. |
| As compared to a master’s degree, I believe an entry-level PA doctoral degree education will better prepare students in the following areas - Clinical practice skills. |
| As compared to a master’s degree, I believe an entry-level PA doctoral degree education will better prepare students in the following areas - The demands of working as a clinician. |
| As compared to a master’s degree, I believe an entry-level PA doctoral degree education will better prepare. students in the following PA competency areas - Patient-centered practice knowledge (Medical Knowledge). |
| As compared to a master’s degree, I believe an entry-level PA doctoral degree education will better prepare students in the following areas - The readiness for team based and collaborative patient care. |
| As compared to a master’s degree, I believe an entry-level PA doctoral degree education will better prepare students in the following areas - Being up-to-date in new or innovative evidence-based practice. |
| As compared to a master’s degree, I believe an entry-level PA doctoral degree education will better prepare students in the following areas - Use of theory in practice. |
| As compared to a master’s degree, I believe an entry-level PA doctoral degree education will better prepare students in the following PA competency areas - Cultural humility. |
| As compared to a master’s degree, I believe an entry-level PA doctoral degree education will better prepare students in the following PA competency areas - Professional and legal aspects of health care. |
| As compared to a master’s degree, I believe an entry-level PA doctoral degree education will better prepare students in the following PA competency areas - Health care finance and systems. |
| As compared to a master’s degree, I believe an entry-level PA doctoral degree education will better prepare students in the following PA competency areas - Society and population health. |
| As compared to a master’s degree, I believe an entry-level PA doctoral degree education will better prepare students in the following PA competency areas - Health literacy and communication. |
| As compared to a master’s degree, I believe an entry-level PA doctoral degree education will better prepare students in the following PA competency areas - Interprofessional collaborative practice and leadership. |
| As compared to a master’s degree, I believe an entry-level PA doctoral degree education will better prepare students in the following PA competency areas - Ongoing Professional development. |
| Some people believe that the current number of credits, depth, and breadth of PA training offered in most PA programs to date are sufficient for a doctoral degree. How strongly do you agree or disagree with this statement? |
| Original response options: *from extremely unlikely to extremely likely* |
| How likely will the following prerequisites be impacted if the PA profession adopts an entry-level PA doctoral degree? – GPA. |
| How likely will the following prerequisites be impacted if the PA profession adopts an entry-level PA doctoral degree? – GRE. |
| How likely will the following prerequisites be impacted if the PA profession adopts an entry-level PA doctoral degree? - Pre-requisite courses (statistics, basic sciences). |
| How likely will the following prerequisites be impacted if the PA profession adopts an entry-level PA doctoral degree? - Prior clinical experience. |
| How likely will the following prerequisites be impacted if the PA profession adopts an entry-level PA doctoral degree? - Entrance examination (ie PCAT). |
| How likely an entry-level PA doctoral degree will - Confuse the patient. |
| How likely an entry-level PA doctoral degree will - Change the certification process. |
| How likely an entry-level PA doctoral degree will - Change the recertification process. |
| How likely an entry-level PA doctoral degree will - Change accreditation standards. |
| How likely an entry-level PA doctoral degree will - Shift practice setting to urban, rural or underserved locations. |
| How likely an entry-level PA doctoral degree will - Shift practice setting to primary care. |
| How likely an entry-level PA doctoral degree will - Increase diversity. |
| How likely an entry-level PA doctoral degree will - Enhance scope of practice. |
